# Dichloridobis[3-meth­oxy­methyl-4-phenyl-5-(2-pyrid­yl)-4*H*-1,2,4-triazole-κ^2^ *N* ^1^,*N* ^5^]chromium(III) chloride

**DOI:** 10.1107/S1600536811039328

**Published:** 2011-10-05

**Authors:** Xiaofei Jin, Zuoxiang Wang, Shouping Cao

**Affiliations:** a School of Chemistry and Engineering, Southeast University, Nanjing 211189, People’s Republic of China

## Abstract

In the title complex, [CrCl_2_(C_15_H_14_N_4_O)_2_]Cl, the Cr^III^ atom is located on a twofold rotation axis and is coordinated by two *N*,*N*′-bidentate triazole derivatives and two chloride ions in a distorted octa­hedral CrN_2_N′_2_Cl_2_ geometry. One of the two independent Cl^−^ counter-anions sits on a special position (site symmetry 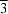.) and is fully occupied, whereas the other is disordered around a twofold rotation axis over two positions in a 2:3 ratio.

## Related literature

For general background to the coordination chemistry of 1,2,4-triazole derivatives, see: Koningsbruggen *et al.* (1997 ▶); Garcia *et al.* (1999 ▶); Klingele & Brooker (2003 ▶); Matsukizono *et al.* (2008 ▶); Suksrichavalit *et al.* (2009 ▶); Rubio *et al.* (2011 ▶). For their biological activity, see: Tozkoparan *et al.* (2000 ▶); Grenman *et al.* (2003 ▶); Alagarsamy *et al.* (2008 ▶); Isloor *et al.* (2009 ▶).
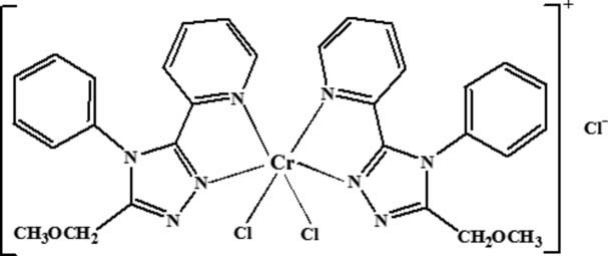


## Experimental

### 

#### Crystal data

[CrCl_2_(C_15_H_14_N_4_O)_2_]Cl
*M* *_r_* = 690.95
Hexagonal, 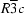

*a* = 20.8852 (12) Å
*c* = 37.620 (4) Å
*V* = 14211.1 (19) Å^3^
*Z* = 18
Mo *K*α radiation
μ = 0.66 mm^−1^
*T* = 296 K
0.17 × 0.13 × 0.12 mm

#### Data collection

Bruker APEXII CCD diffractometer
Absorption correction: multi-scan (*SADABS*; Sheldrick, 2003 ▶) *T*_min_ = 0.896, *T*_max_ = 0.925
32255 measured reflections
2785 independent reflections
2243 reflections with *I* > 2σ(*I*)
*R*_int_ = 0.052

#### Refinement

*R*[*F* ^2^ > 2σ(*F* ^2^)] = 0.039
*wR*(*F* ^2^) = 0.119
*S* = 1.10
2785 reflections
213 parameters
18 restraints
H-atom parameters constrained
Δρ_max_ = 0.68 e Å^−3^
Δρ_min_ = −0.31 e Å^−3^

### 

Data collection: *APEX2* (Bruker, 2005 ▶); cell refinement: *SAINT* (Bruker, 2005 ▶); data reduction: *SAINT*; program(s) used to solve structure: *SHELXS97* (Sheldrick, 2008 ▶); program(s) used to refine structure: *SHELXL97* (Sheldrick, 2008 ▶); molecular graphics: *XP* in *SHELXTL* (Sheldrick, 2008 ▶); software used to prepare material for publication: *SHELXTL*.

## Supplementary Material

Crystal structure: contains datablock(s) I, global. DOI: 10.1107/S1600536811039328/wm2515sup1.cif

Structure factors: contains datablock(s) I. DOI: 10.1107/S1600536811039328/wm2515Isup2.hkl

Additional supplementary materials:  crystallographic information; 3D view; checkCIF report

## Figures and Tables

**Table 1:** Selected bond lengths (Å)

Cr1—N1	2.040 (2)
Cr1—N4	2.0949 (19)
Cr1—Cl2^i^	2.2746 (7)

Symmetry code: (i) 
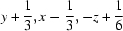
.

**Fig. 1.**
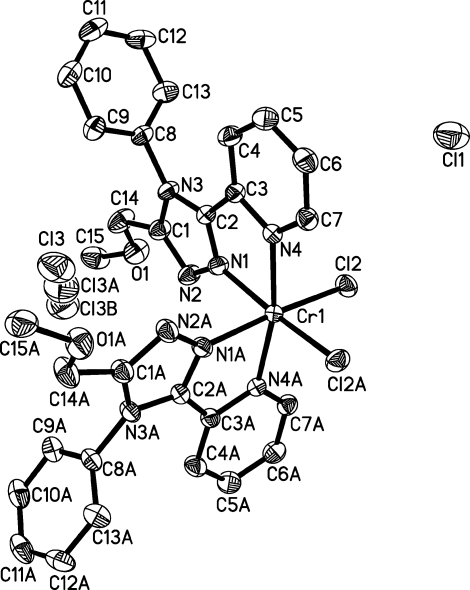
The molecular structure of the title compound with the atomic labelling. Displacement ellipsoids are shown at 30% probability level. [Symmetry code: A) y + 1/3, x - 1/3, -z + 1/6.]

## supplementary crystallographic information

### Comment

As the 1,2,4-triazole ring posesses strong electron donors, the coordination chemistry of 1,2,4-triazoles employed as ligand is widely studied (Koningsbruggen *et al.*, 1997; Garcia *et al.*, 1999; Klingele & Brooker, 2003; Matsukizono *et al.*, 2008; Suksrichavalit *et al.*, 2009; Rubio *et al.*, 2011). Moreover, some 1,2,4-triazole compounds show biological activities (Tozkoparan *et al.*, 2000; Grenman *et al.*, 2003; Alagarsamy *et al.*, 2008; Isloor *et al.*, 2009). We report here the crystal structure analysis of the title compound, [Cr(C_15_H_14_N_4_O)_2_Cl_2_]Cl.

In the title compouund, the chromium(III) atom is coordinated by two chelating 3-(methoxymethyl)-4-phenyl-5-(2-pyridyl)-4*H*-1,2,4- triazole ligands and two chloride anions in a distorted octahedral geometry with a CrN_2_N'_2_Cl_2_ coordination set. The central Cr^III^ atom is located on a special position (site symmetry .2). The dihedral angle between the 1,2,4-triazole ring and the phenyl ring is 83.28 (16) °. One of the two non-coordinating Cl^-^ counter is located on a special position (site symmetry 3.) whereas the other shows disorder around a twofold rotation axis.

### Experimental

To a warm solution of 0.798 g of 3-(methoxymethyl)-4-phenyl-5-(2-pyridyl)-4*H*-1,2,4- triazole (3 mmol) in 20 ml ethanol, 0.399 g of CrCl_3_^.^6H_2_O (1.5 mmol) were added. The filtrate was left to stand at room temperature for several days. The dark purple product was collected, and single crystals suitable for X-ray diffraction were selected.

### Refinement

Positional parameters of all the H atoms were calculated geometrically and were allowed to ride on the C atoms to which they are bonded, riding with C—H = 0.93 Å (aromatic), 0.96 Å (methyl) and 0.97 Å (methylene), with U_iso_(H) = 1.2 or 1.5 times U_eq_(C). Atom CL3 is positionally and occupationally disordered around a twofold rotation axis. One part (Cl3A, site occupation factor 0.168 (10)) sits on the twofold axis whereas the other part (Cl3) is 1.014 (19) Å away from this atom with a s.o.f. of 0.249 (5). To achieve an electroneutral compound, the overall occuipancy of the two Cl3 atoms was restrained to 0.3.

### Crystal data

**Table:**

[CrCl_2_(C_15_H_14_N_4_O)_2_]Cl	*D*_x_ = 1.453 Mg m^−^^3^
*M**_r_* = 690.95	Mo *K*α radiation, λ = 0.71073 Å
Hexagonal, *R*3*c*	Cell parameters from 9999 reflections
Hall symbol: -R 3 2"c	θ = 2.4–21.0°
*a* = 20.8852 (12) Å	µ = 0.66 mm^−^^1^
*c* = 37.620 (4) Å	*T* = 296 K
*V* = 14211.1 (19) Å^3^	Octahedral, purple
*Z* = 18	0.17 × 0.13 × 0.12 mm
*F*(000) = 6390	

### Data collection

**Table:**

Bruker APEXII CCD diffractometer	2785 independent reflections
Radiation source: fine-focus sealed tube	2243 reflections with *I* > 2σ(*I*)
graphite	*R*_int_ = 0.052
ω scans	θ_max_ = 25.0°, θ_min_ = 2.0°
Absorption correction: multi-scan (*SADABS*; Sheldrick, 2003)	*h* = −24→24
*T*_min_ = 0.896, *T*_max_ = 0.925	*k* = −24→24
32255 measured reflections	*l* = −44→42

### Refinement

**Table:**

Refinement on *F*^2^	Primary atom site location: structure-invariant direct methods
Least-squares matrix: full	Secondary atom site location: difference Fourier map
*R*[*F*^2^ > 2σ(*F*^2^)] = 0.039	Hydrogen site location: inferred from neighbouring sites
*wR*(*F*^2^) = 0.119	H-atom parameters constrained
*S* = 1.10	*w* = 1/[σ^2^(*F*_o_^2^) + (0.072*P*)^2^ + 5.702*P*] where *P* = (*F*_o_^2^ + 2*F*_c_^2^)/3
2785 reflections	(Δ/σ)_max_ = 0.002
213 parameters	Δρ_max_ = 0.68 e Å^−^^3^
18 restraints	Δρ_min_ = −0.31 e Å^−^^3^

### Special details

**Table:**

Geometry. All e.s.d.'s (except the e.s.d. in the dihedral angle between two l.s. planes) are estimated using the full covariance matrix. The cell e.s.d.'s are taken into account individually in the estimation of e.s.d.'s in distances, angles and torsion angles; correlations between e.s.d.'s in cell parameters are only used when they are defined by crystal symmetry. An approximate (isotropic) treatment of cell e.s.d.'s is used for estimating e.s.d.'s involving l.s. planes.
Refinement. Refinement of *F*^2^ against ALL reflections. The weighted *R*-factor *wR* and goodness of fit *S* are based on *F*^2^, conventional *R*-factors *R* are based on *F*, with *F* set to zero for negative *F*^2^. The threshold expression of *F*^2^ > σ(*F*^2^) is used only for calculating *R*-factors(gt) *etc*. and is not relevant to the choice of reflections for refinement. *R*-factors based on *F*^2^ are statistically about twice as large as those based on *F*, and *R*- factors based on ALL data will be even larger.

### Fractional atomic coordinates and isotropic or equivalent isotropic displacement parameters (Å^2^)

**Table:**

	*x*	*y*	*z*	*U*_iso_*/*U*_eq_	Occ. (<1)
C1	0.91830 (15)	0.47477 (15)	0.03037 (7)	0.0461 (6)	
Cl1	1.0000	1.0000	0.0000	0.0778 (6)	
Cl2	1.12055 (4)	0.71828 (4)	0.055158 (18)	0.0496 (2)	
Cl3	0.7438 (5)	0.4643 (3)	0.07589 (15)	0.109 (2)	0.249 (5)
Cl3A	0.7727 (7)	0.4394 (7)	0.0833	0.113 (5)	0.168 (10)
Cr1	1.01043 (2)	0.67710 (2)	0.0833	0.03157 (19)	
N1	0.96416 (11)	0.58203 (11)	0.05415 (5)	0.0386 (5)	
O1	0.94481 (13)	0.37801 (11)	0.03630 (6)	0.0668 (6)	
C2	0.92596 (13)	0.58194 (13)	0.02635 (6)	0.0363 (6)	
N2	0.96048 (13)	0.51506 (11)	0.05722 (6)	0.0467 (6)	
C3	0.92437 (13)	0.64891 (13)	0.01735 (6)	0.0369 (6)	
N3	0.89587 (12)	0.51482 (11)	0.01048 (5)	0.0408 (5)	
C4	0.89089 (15)	0.65933 (16)	−0.01212 (7)	0.0484 (7)	
H4	0.8639	0.6207	−0.0278	0.058*	
N4	0.96271 (10)	0.70375 (10)	0.04105 (5)	0.0345 (5)	
C5	0.89817 (16)	0.72790 (16)	−0.01779 (8)	0.0533 (7)	
H5	0.8766	0.7362	−0.0376	0.064*	
C6	0.93721 (16)	0.78347 (16)	0.00588 (8)	0.0504 (7)	
H6	0.9428	0.8301	0.0023	0.060*	
C7	0.96843 (14)	0.76974 (14)	0.03532 (7)	0.0421 (6)	
H7	0.9942	0.8076	0.0516	0.051*	
C8	0.84783 (14)	0.49011 (14)	−0.02045 (7)	0.0413 (6)	
C9	0.77403 (16)	0.46556 (15)	−0.01567 (8)	0.0521 (7)	
H9	0.7551	0.4630	0.0070	0.062*	
C10	0.72852 (18)	0.44481 (16)	−0.04488 (9)	0.0598 (8)	
H10	0.6785	0.4287	−0.0421	0.072*	
C11	0.7567 (2)	0.44776 (17)	−0.07810 (9)	0.0631 (9)	
H11	0.7258	0.4332	−0.0978	0.076*	
C12	0.8305 (2)	0.47214 (18)	−0.08231 (8)	0.0617 (9)	
H12	0.8491	0.4741	−0.1050	0.074*	
C13	0.87821 (17)	0.49400 (17)	−0.05344 (7)	0.0538 (7)	
H13	0.9283	0.5105	−0.0562	0.065*	
C14	0.8939 (2)	0.39585 (17)	0.02282 (9)	0.0651 (9)	
H14A	0.8459	0.3644	0.0336	0.078*	
H14B	0.8890	0.3874	−0.0026	0.078*	
C15	0.9149 (2)	0.30032 (18)	0.04022 (9)	0.0744 (10)	
H15A	0.9495	0.2911	0.0527	0.112*	
H15B	0.9054	0.2776	0.0172	0.112*	
H15C	0.8695	0.2799	0.0534	0.112*	

### Atomic displacement parameters (Å^2^)

**Table:**

	*U*^11^	*U*^22^	*U*^33^	*U*^12^	*U*^13^	*U*^23^
C1	0.0529 (16)	0.0402 (14)	0.0461 (15)	0.0241 (13)	−0.0090 (12)	−0.0049 (12)
Cl1	0.0920 (10)	0.0920 (10)	0.0494 (11)	0.0460 (5)	0.000	0.000
Cl2	0.0408 (4)	0.0593 (4)	0.0460 (4)	0.0230 (3)	0.0107 (3)	0.0075 (3)
Cl3	0.162 (5)	0.081 (3)	0.074 (3)	0.053 (3)	0.008 (3)	−0.008 (2)
Cl3A	0.121 (5)	0.121 (5)	0.089 (6)	0.053 (5)	0.013 (4)	−0.013 (4)
Cr1	0.0317 (2)	0.0317 (2)	0.0285 (3)	0.0137 (2)	−0.00026 (11)	0.00026 (11)
N1	0.0433 (12)	0.0364 (11)	0.0357 (11)	0.0195 (10)	−0.0063 (9)	−0.0019 (9)
O1	0.0815 (15)	0.0456 (12)	0.0795 (15)	0.0364 (11)	−0.0167 (12)	−0.0035 (10)
C2	0.0354 (13)	0.0355 (13)	0.0348 (13)	0.0153 (11)	−0.0035 (10)	−0.0025 (10)
N2	0.0570 (14)	0.0368 (12)	0.0464 (13)	0.0235 (11)	−0.0147 (11)	−0.0052 (10)
C3	0.0333 (13)	0.0402 (14)	0.0353 (13)	0.0170 (11)	−0.0017 (10)	0.0000 (11)
N3	0.0444 (12)	0.0385 (12)	0.0380 (12)	0.0195 (10)	−0.0119 (9)	−0.0077 (9)
C4	0.0507 (16)	0.0506 (16)	0.0436 (15)	0.0250 (13)	−0.0131 (12)	0.0004 (12)
N4	0.0347 (10)	0.0347 (11)	0.0320 (11)	0.0156 (9)	0.0018 (8)	0.0025 (8)
C5	0.0549 (17)	0.0564 (18)	0.0526 (17)	0.0309 (15)	−0.0099 (14)	0.0084 (14)
C6	0.0580 (17)	0.0441 (15)	0.0540 (18)	0.0293 (14)	0.0019 (14)	0.0102 (13)
C7	0.0449 (14)	0.0373 (14)	0.0443 (15)	0.0206 (12)	0.0012 (12)	0.0021 (11)
C8	0.0453 (15)	0.0389 (14)	0.0414 (15)	0.0222 (12)	−0.0119 (12)	−0.0096 (11)
C9	0.0483 (16)	0.0450 (16)	0.0565 (18)	0.0184 (13)	−0.0061 (13)	−0.0083 (13)
C10	0.0513 (17)	0.0476 (17)	0.077 (2)	0.0219 (14)	−0.0214 (16)	−0.0115 (15)
C11	0.076 (2)	0.0477 (17)	0.072 (2)	0.0359 (17)	−0.0394 (18)	−0.0169 (15)
C12	0.096 (3)	0.066 (2)	0.0365 (16)	0.0504 (19)	−0.0136 (16)	−0.0093 (13)
C13	0.0610 (18)	0.0626 (19)	0.0467 (17)	0.0375 (16)	−0.0064 (14)	−0.0079 (14)
C14	0.079 (2)	0.0469 (17)	0.074 (2)	0.0345 (17)	−0.0306 (18)	−0.0187 (15)
C15	0.117 (3)	0.058 (2)	0.056 (2)	0.049 (2)	−0.001 (2)	0.0027 (15)

### Geometric parameters (Å, °)

**Table:**

C1—N2	1.328 (3)	C5—C6	1.363 (4)
C1—N3	1.368 (3)	C5—H5	0.9300
C1—C14	1.489 (4)	C6—C7	1.386 (4)
Cl2—Cr1	2.2747 (7)	C6—H6	0.9300
Cl3—Cl3^i^	2.028 (19)	C7—H7	0.9300
Cr1—N1^i^	2.040 (2)	C8—C9	1.371 (4)
Cr1—N1	2.040 (2)	C8—C13	1.378 (4)
Cr1—N4^i^	2.0948 (19)	C9—C10	1.374 (4)
Cr1—N4	2.0949 (19)	C9—H9	0.9300
Cr1—Cl2^i^	2.2746 (7)	C10—C11	1.370 (4)
N1—C2	1.315 (3)	C10—H10	0.9300
N1—N2	1.367 (3)	C11—C12	1.369 (5)
O1—C14	1.387 (4)	C11—H11	0.9300
O1—C15	1.425 (4)	C12—C13	1.387 (4)
C2—N3	1.355 (3)	C12—H12	0.9300
C2—C3	1.456 (3)	C13—H13	0.9300
C3—N4	1.353 (3)	C14—H14A	0.9700
C3—C4	1.384 (3)	C14—H14B	0.9700
N3—C8	1.452 (3)	C15—H15A	0.9600
C4—C5	1.379 (4)	C15—H15B	0.9600
C4—H4	0.9300	C15—H15C	0.9600
N4—C7	1.340 (3)		
			
N2—C1—N3	110.5 (2)	C6—C5—H5	120.3
N2—C1—C14	126.8 (2)	C4—C5—H5	120.3
N3—C1—C14	122.7 (2)	C5—C6—C7	119.3 (3)
N1^i^—Cr1—N1	87.33 (12)	C5—C6—H6	120.4
N1^i^—Cr1—N4^i^	78.06 (8)	C7—C6—H6	120.4
N1—Cr1—N4^i^	93.14 (8)	N4—C7—C6	122.1 (2)
N1^i^—Cr1—N4	93.15 (8)	N4—C7—H7	118.9
N1—Cr1—N4	78.06 (8)	C6—C7—H7	118.9
N4^i^—Cr1—N4	167.94 (10)	C9—C8—C13	122.6 (3)
N1^i^—Cr1—Cl2^i^	90.84 (6)	C9—C8—N3	118.4 (2)
N1—Cr1—Cl2^i^	172.01 (6)	C13—C8—N3	119.0 (2)
N4^i^—Cr1—Cl2^i^	94.08 (5)	C8—C9—C10	119.0 (3)
N4—Cr1—Cl2^i^	94.29 (6)	C8—C9—H9	120.5
N1^i^—Cr1—Cl2	172.01 (6)	C10—C9—H9	120.5
N1—Cr1—Cl2	90.83 (6)	C11—C10—C9	120.1 (3)
N4^i^—Cr1—Cl2	94.29 (6)	C11—C10—H10	120.0
N4—Cr1—Cl2	94.07 (5)	C9—C10—H10	120.0
Cl2^i^—Cr1—Cl2	92.02 (4)	C12—C11—C10	120.1 (3)
C2—N1—N2	110.0 (2)	C12—C11—H11	120.0
C2—N1—Cr1	114.91 (16)	C10—C11—H11	120.0
N2—N1—Cr1	135.02 (15)	C11—C12—C13	121.4 (3)
C14—O1—C15	112.6 (3)	C11—C12—H12	119.3
N1—C2—N3	108.6 (2)	C13—C12—H12	119.3
N1—C2—C3	119.3 (2)	C8—C13—C12	116.9 (3)
N3—C2—C3	132.1 (2)	C8—C13—H13	121.5
C1—N2—N1	105.3 (2)	C12—C13—H13	121.6
N4—C3—C4	121.7 (2)	O1—C14—C1	110.1 (2)
N4—C3—C2	111.8 (2)	O1—C14—H14A	109.6
C4—C3—C2	126.4 (2)	C1—C14—H14A	109.6
C2—N3—C1	105.6 (2)	O1—C14—H14B	109.6
C2—N3—C8	127.0 (2)	C1—C14—H14B	109.6
C1—N3—C8	127.4 (2)	H14A—C14—H14B	108.2
C5—C4—C3	119.0 (3)	O1—C15—H15A	109.5
C5—C4—H4	120.5	O1—C15—H15B	109.5
C3—C4—H4	120.5	H15A—C15—H15B	109.5
C7—N4—C3	118.4 (2)	O1—C15—H15C	109.5
C7—N4—Cr1	125.86 (17)	H15A—C15—H15C	109.5
C3—N4—Cr1	115.74 (15)	H15B—C15—H15C	109.5
C6—C5—C4	119.4 (3)		

Symmetry codes: (i) *y*+1/3, *x*−1/3, −*z*+1/6.
